# Effectiveness of Multidisciplinary “Case Management” Approaches to the Management of Patients Who Make High Use of the Emergency Ambulance Service: A Quasi-experimental Evaluation Using Linked Routine Records

**DOI:** 10.1016/j.acepjo.2026.100459

**Published:** 2026-07-13

**Authors:** Alan Watkins, Rabeea’h Waseem Aslam, Timothy Driscoll, Adrian Edwards, Bethan Edwards, Bridie Angela Evans, Theresa Foster, Rachael Fothergill, Penny Gripper, Imogen M. Gunson, Ann John, Ashra Khanom, Robin Petterson, Alison Porter, Nigel Rees, Andy Rosser, Jason Scott, Bernadette Sewell, Anna Tee, Helen Snooks

**Affiliations:** 1 Faculty of Medicine, Health & Life Sciences, Swansea University, Swansea, UK; 2 School of Social Sciences, Cardiff University, Cardiff, UK; 3 Division of Population Medicine, Cardiff University, Cardiff, UK; 4 School of Mental Health & Psychological Sciences, King’s College London, London, UK; 5 East of England Ambulance Service NHS Trust, Melbourn, Cambridgeshire, UK; 6 London Ambulance Service Trust, London, UK; 7 Public contributor, Swansea, UK; 8 West Midlands Ambulance Service University NHS Foundation Trust, Brierly Hill, West Midlands, UK; 9 Welsh Ambulance Services NHS Trust, Cwmbran, Gwent, UK; 10 Department of Social Work, Education & Community Wellbeing, Northumbria University, Newcastle upon Tyne, UK; 11 Independent Research Consultant, Carmarthen, Carmarthenshire, UK

**Keywords:** case management, cohort study, electronic health record, emergency ambulance service, frequent caller, natural experiment

## Abstract

**Objectives:**

A minority of people make high use of emergency ambulance services. Some UK ambulance services have deployed multidisciplinary case management for these patients. This approach extends usual (within-service) care provision to include input from other agencies such as social care. We aimed to evaluate the effectiveness of case management compared with usual care.

**Methods:**

Natural experiment retrospective cohort study with patients from 4 UK ambulance services. We accessed anonymized electronic health records for patient cohorts identified by ambulance services. We compared patient-level outcomes based on mortality and emergency health contacts within 6 months of patient eligibility for case management. Cohorts were compared using logistic regression models, adjusting for ambulance service, patient characteristics, and service use in the 6 months before patients became eligible.

**Results:**

We found no differences between case management (intervention; n = 550) and usual care (control; n = 633) cohorts for our primary outcome, defined as death or any emergency health contact within 6 months. Nearly all patients recorded at least 1 such contact: 526 of 550 (95.6%) in the intervention cohort, compared with 601 of 633 (94.9%) in the control (adjusted odds ratio 1.159; 95% CI, 0.595-2.255). Mortality at 6 months was high: 58 of 550 (10.5%) in the intervention cohort and 89 of 633 (14.1%) in the control cohort.

**Conclusion:**

We found no evidence for the clinical effectiveness of multidisciplinary case management over within-service usual care. Study patients had a high risk of death within 6 months of eligibility for case management, and almost all made at least 1 recorded emergency health contact in that period.

**The Bottom Line**

This study evaluated the effectiveness of a multidisciplinary case management approach to the care of patients who make high use of emergency ambulance services. We analyzed n = 1183 patient-level outcomes for the 6 months following eligibility for case management in cohorts of patients identified in 4 UK ambulance services. We found no evidence for the clinical effectiveness of case management over usual in-service care. Study patients had a high risk of death (12.4%) within 6 months of eligibility for case management, and almost all (95.3%) made at least 1 recorded emergency health contact in that period.

## 1 Introduction

### 1.1 Background

In common with other middle and upper-income countries, emergency ambulance services in the UK are under sustained pressure and are required to focus resources on people with urgent or life-threatening conditions.^1^ Emergency ambulance services, an integral part of the free-to-use National Health Service (NHS) and managed on a regional basis, respond to over a million calls per month to the emergency 999 telephone number. Around half result in patient conveyance to the emergency department (ED, 48.9%) or elsewhere (4.5%); a quarter (28.4%) are resolved by an attending clinician (paramedic or emergency medical technician) without conveyance, and the remainder (18.1%) are resolved over the phone.^2^

A small minority of people make high use of emergency ambulance services. These individuals are often patients with complex needs and potentially vulnerable to mental health or other crises.^3^ They are more likely to be under socioeconomic stress, live alone, experience mental health problems, have a history of trauma or abuse, live with chronic conditions, or fall.^4^, ^5^, ^6^, ^7^ People who make high use of emergency ambulance and ED services frequently are known to have higher short-term mortality rates than those who seek care less often.^6^, ^7^, ^8^, ^9^

Usual care for patients identified as high users, as defined by national criteria, generally consists of ambulance services discouraging them from calling again or flagging the patient to limit future responses.^10^ This may leave callers’ needs unresolved and shift unmet demand from one part of the health and social care system to another, with concomitant resource implications.^11^

### 1.2 Importance

Emergency ambulance services may not have resources to respond to all who call. People who make frequent calls to the emergency ambulance service may not have their clinical needs resolved through these contacts, sometimes with mismatched expectations compared with those of ambulance service staff.^5^

In some areas, some UK ambulance services, supported by policymakers at national and local levels, have introduced multidisciplinary cross-agency approaches with input from emergency, primary, and social care. This is known as ‘case management’ and is intended to reduce short-term mortality and emergency health care contacts. However, we lack evidence evaluating this approach for the care of patients who make high use of emergency ambulance services.

### 1.3 Goals of This Investigation

We designed the strategies to manage emergency ambulance telephone callers with sustained high needs: an evaluation using linked data (STRETCHED) protocol study to evaluate the effectiveness and safety of multidisciplinary cross-agency case management for the care of people who frequently call the emergency ambulance service compared with usual (within-service) care and to describe the epidemiology of this patient group.^12^

## 2 Methods

### 2.1 Study Design

In this natural experiment retrospective cohort study, we took advantage of variations in practice between administrative units (Local Health Boards [LHBs] in Wales and Clinical Commissioning Groups [CCGs] in England) within ambulance service catchment areas to evaluate case management approaches being implemented within some UK ambulance services.^12^

STRETCHED protocol included 8 sites across 4 UK ambulance services, with 1 case management (‘intervention’) and 1 usual care (‘control’) site in each service. The East of England Ambulance Service NHS Trust (EEAS), London Ambulance Service NHS Trust (LAS), Welsh Ambulance Services University NHS Trust (WAST), and West Midlands Ambulance Service University NHS Foundation Trust (WMAS) were approached following a survey of practice across the UK, which identified where both case management and usual models of care were in place in different areas within an ambulance service’s catchment area.^11^ Two other ambulance services met our criteria for participation; these were excluded after initial engagement identified concerns over data availability and cohort size.

### 2.2 Setting

UK ambulance services cover large areas, each serving a population of between 3 and 9 million people across a mixture of urban and rural locations. They provide a range of emergency responses, including telephone advice, attendance of an emergency vehicle for face-to-face assessment by a paramedic or emergency medical technician, and referral to another health care provider or conveyance to hospital when judged clinically appropriate. This care, free at the point of use, is usually initiated by dialing the UK’s nationwide emergency response system. Calls are then routed to an appropriate service, including the local ambulance service. Ambulance service responses will reflect, inter alia, local services available, which may vary by LHB or CCG area.

Population characteristics for the 8 STRETCHED protocol study sites are summarized in Supplementary Appendix 1; sites within each ambulance service area are broadly similar, with clear differences in some characteristics across ambulance service areas. The 8 study sites together comprise a representative cross-section of the UK population.

### 2.3 Participants

Our study population was adults (aged 18+ years) living in study sites who met national criteria for classification as a high user (“frequent caller”) by ambulance services (≥5 emergency ambulance service calls in 1 month, or ≥12 calls in 3 months) during 2018. Two ambulance services (AS1 and AS2) also included patients classified as frequent callers at the start of 2018. Patient management depended on their home address—patients living in specific, defined areas (determined by local commissioning decisions) were eligible for case management; patients outside those areas received usual care. Control sites were selected by ambulance services to be as similar as possible to the intervention site.

Based on data provided by sites during study design, we expected to identify 158 eligible patients per study site (316 per ambulance service; 632 per arm; 1264 cases in total). We assumed that no >5% of cases would be lost to follow-up (eg, unavailability of patient-level data), resulting in an analysable data set comprising 300 patients per ambulance service (n = 1200), with 90% power to detect a standardized statistical effect of 0.2 at the 5% significance level.

### 2.4 Intervention/Exposure

The exposure of interest was eligibility for case management, a multidisciplinary cross-agency approach which extends usual (within-service) care and is intended to provide a wider network of tailored support for patients, intended to reduce short-term mortality and emergency health care contacts. A more detailed comparison between case management and usual care is given in Supplementary Appendix 2.

### 2.5 Measurements

We obtained dates of 999 calls from ambulance service electronic health record (EHR) systems. We then determined the ‘index’ date each patient first became eligible for inclusion in the STRETCHED protocol (ie, made 5 or more calls in the previous month or 12 or more calls in the previous 3 months) and defined baseline (the 6 months immediately preceding the index date) and follow-up periods (6 months from the index date) for each patient. We then calculated the number of 999 calls in the baseline and follow-up periods, and whether there was any point during the follow-up period that the patient had not made 5 or more calls within the previous month.

We obtained dates of ED presentations; hospital admissions (including whether planned or unplanned); outpatient appointments; and demographics data from national hospital data repositories for England and Wales. For each patient, we determined whether death was recorded within 6 months of the index date and calculated the number of ED presentations; emergency (ie, unplanned) hospital admissions; elective (ie, planned) hospital admissions; and outpatient appointments within the baseline and follow-up periods. Patient demographics included age in years on the index date (derived from month and year of birth), gender (patient self-declared), and ethnicity (dichotomized to White/non-White because of low counts in some groups). We also obtained Townsend deprivation score quintiles, a widely used and validated measure of material deprivation, from the lower super output area (LSOA; comprising between 400 and 1200 households) of a patient’s home address.^13^

### 2.6 Outcomes

Our primary outcome was a patient-level binary composite indicator of whether any of mortality, an emergency hospital admission, an ED attendance, or an emergency ambulance service call occurred within the 6-month follow-up period. This composite outcome and follow-up period reflected the main objectives of case management; their selection followed discussions with both ambulance services and patients, including the development of a logic model for case management.

We compared components individually and cumulatively according to severity (first, mortality; next, emergency hospital admissions; then, ED attendances; and finally, ambulance service calls), because all components were judged as important and potentially affected by case management and possibly in different directions with shifting demand.

Secondary outcomes were based on the numbers of emergency (unplanned) hospital admissions recorded; ED attendances recorded; emergency ambulance service calls recorded; elective (planned) hospital appointments; outpatient appointments; and whether the patient no longer met the ‘frequent caller’ criteria at any point within follow-up—and, if so, whether they subsequently met the criteria again; and whether any adverse events (injuries, serious medical emergencies, and police arrests) were recorded.

### 2.6 Analysis

We summarized patient recruitment using a Consolidated Standards of Reporting Trials (CONSORT) flowchart, with descriptive data summaries for patient characteristics and baseline characteristics (counts and percentages for categoric variables, means and standard deviations for continuous variables, or medians and quartiles where nonnormally distributed).^14^

We used logistic regression models for binary outcomes (mortality, whether further health care contacts were recorded, whether patients no longer met the ‘frequent caller’ criteria, and whether they subsequently met the criteria again) and generalized linear models for counts (number of health care contacts recorded). Models were adjusted for ambulance service, patient characteristics (age in years; gender; ethnicity; Townsend deprivation quintiles) and baseline service use (number of emergency hospital admissions; ED presentations; and emergency ambulance calls). We also adjusted for the number of baseline elective admissions and outpatient appointments for the corresponding secondary outcome. These adjustment factors were proposed and confirmed during study design. We did not adjust for multiplicity, as the primary outcome had a prespecified order of analysis. Modeling assumptions were assessed using variance inflation factors; goodness of fit was assessed using standard metrics including the Hosmer-Lemeshow test, Cox and Snell pseudo-R^2^, the Box-Tidwell test; and graphically using residual and leverage plots.

Use of EHR means that ambulance service and hospital contact data are assumed to be complete. Missing demographic characteristic data were managed using multiple imputation models, incorporating age (constrained to 18-110), gender, ethnicity (dichotomized to Caucasian/non-Caucasian), and Townsend deprivation quintile. Ambulance service, site, and study arm were included as indicator variables.

All comparisons were based on treatment allocated (the ‘intention to treat’ principle) using 2-sided tests evaluated at the 5% significance level. Comparisons are presented as odds ratios (ORs; logistic regression) or incident rate ratios (generalized linear models) with 95% confidence intervals. Analysis was conducted using IBM SPSS version 29 hosted within the SAIL Gateway, a Trusted Research Environment maintained by Swansea University.^15^

### 2.7 Ethical Approval

We received UK Health Research Authority approval for the STRETCHED protocol, based on a favorable opinion from an NHS Research Ethics Committee (19/WA/0216) and Confidential Advisory Group (CAG; 19/CAG/0195) support for accessing patient data without consent.

### 2.8 Trial Registration

STRETCHED protocol was registered as researchregistry7895 with researchregistry.com.

### 2.9 Patient and Public Involvement

Throughout the design, conduct and dissemination of the STRETCHED protocol, we involved public contributors with experience of accessing emergency ambulance services and the complex health conditions experienced by people making frequent emergency calls.^12^ Various fora - strategic, managerial, oversight and implementation - facilitated contributions to STRETCHED protocol from individuals with diverse backgrounds and perspectives. Public contributors ensured service users’ issues remained in focus - for instance, over the importance of language, highlighting that the term ‘frequent caller’ can be perceived as labeling and that it is preferable to refer to people who make frequent calls.

## 3 Results

### 3.1 Cohort Identification

Researchers at participating ambulance services retrospectively identified a total of 1757 eligible patients (1103 intervention and 654 control). There was considerable variation in the numbers of patients identified by ambulance service and study arm; most noticeably, over half the intervention patients came from 1 site (AS4; Fig).

**Figure:**
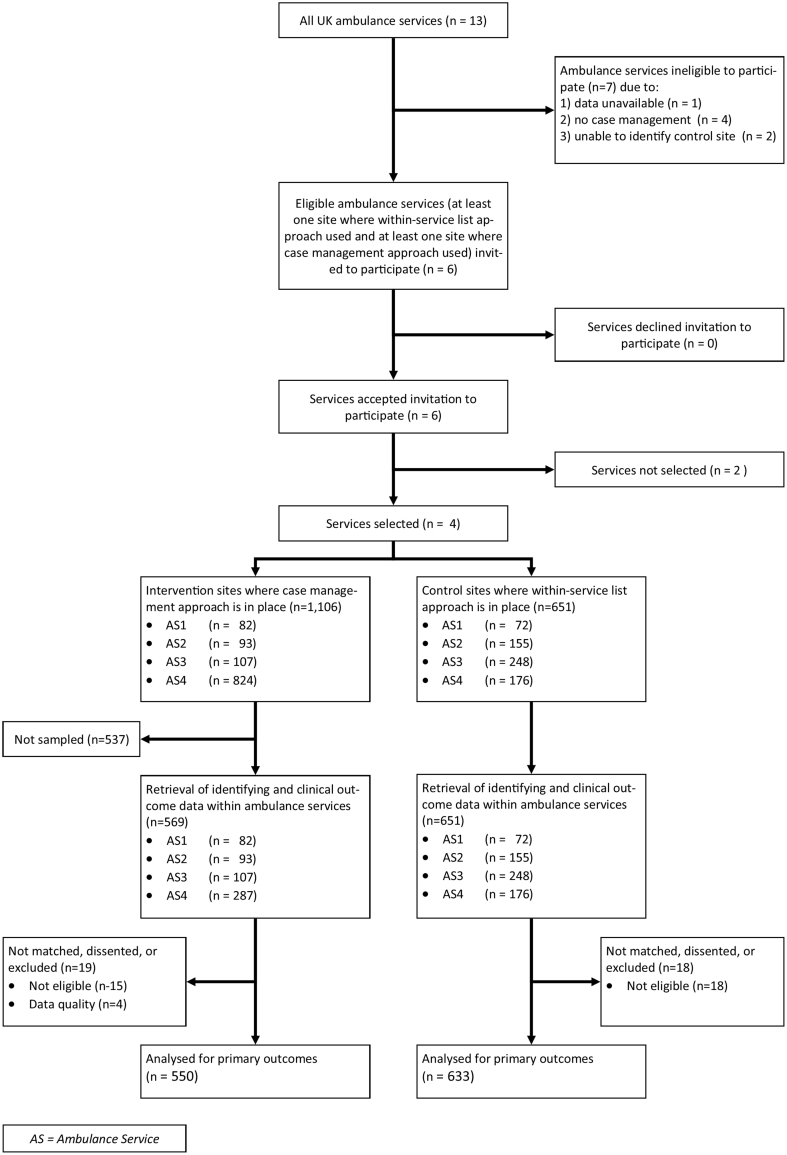
STRETCHED patient flow diagram.

Data including patient identifiers, demographic characteristics, emergency ambulance service calls, ambulance dispatches, serious case reviews, and (where available) data on arrests and convictions were extracted for 1220 of these patients (569 intervention and 651 control) and linked with EHR from national data repositories (NHS England for England; SAIL Databank for Wales). No patients were lost in the linkage process.

In total, 37 patients (19 intervention and 18 control) were excluded during data cleaning, an attrition rate of 3%, with most (33 patients; 15 intervention and 18 control) due to patients not meeting study inclusion criteria. The primary outcome analysis thus included data on 1183 patients (550 intervention and 633 control).

### 3.2 Cohort Characteristics

Overall, the study cohort was relatively old, with a median age of 65 years (lower quartile 45 and upper quartile 81), with slightly more females (51.9%) than males, and was predominantly White (81.7%). Just under two-thirds of patients (65.1%) lived in LSOAs classified in the 2 most socioeconomically deprived quintiles, whereas only 5.2% lived in the least socioeconomically deprived quintile. There are clear differences between demographic profiles for the STRETCHED protocol study population (Table 1) and the corresponding profiles for the general adult populations in study sites (Supplementary Appendix 1).

**Table 1:** Patient demographic characteristics at baseline, by study arm and AS.

Characteristic	Intervention		Control	
Patients identified	N = 550		N = 633	
AS1	82	(14.9%)	72	(11.4%)
AS2	77	(14.0%)	137	(21.6%)
AS3	107	(19.5%)	248	(39.2%)
AS4	284	(51.6%)	176	(27.8%)
Age (y): median (IQR)^a^	(n = 520)		(n = 583)	
Overall	60	(41- 80)	69	(49-82)
AS1	62	(45-82)	58	(47-76)
AS2	50	(38-71)	63	(47-82)
AS3	65	(47-80)	73	(52-83)
AS4	60	(39-81)	69	(48-81)
Gender: n (%) female	(n =4 99)		(n = 572)	
Overall	266/499	(53.3%)	290/572	(50.7%)
AS1	45/76	(59.2%)	30/60	(50.0%)
AS2	26/59	(44.1%)	48/104	(46.2%)
AS3	62/107	(57.9%)	123/248	(49.6%)
AS4	133/257	(51.8%)	89/160	(55.6%)
Ethnicity: n (%) non-White^b^	72/400	(18.0%)	75/412	(18.2%)
Townsend quintile^b^^,^^c^	(n = 504)		(n = 577)	
Q1	28	(5.6%)	28	(4.9%)
Q2	61	(12.1%)	55	(9.5%)
Q3	81	(16.1%)	126	(21.8%)
Q4	99	(19.6%)	189	(32.8%)
Q5	235	(46.6%)	179	(31.0%)

AS, ambulance service

a IQR is presented as lower quartile-upper quartile.

b Patient numbers on ethnicity and deprivation quintiles are not presented by AS to mitigate the risk of inadvertent disclosure from low subgroup counts.

c Townsend UK deprivation score quintiles; Q1 and Q5 are, respectively, the least and most deprived quintiles.

The 2 study arms were reasonably well matched with respect to these demographic characteristics, except for age – intervention patients were generally younger than control counterparts in 3 participating ambulance services (AS2, AS3, and AS4). The proportion of female patients varied from 44.1% (AS2 intervention site) to 59.2% (AS1 intervention site).

In the 6 months before patients became eligible for inclusion in the STRETCHED protocol, emergency ambulance service use and hospital use were similar between arms, with at least 1 emergency hospital admission recorded for just under two-thirds (63.7%) of patients (Table 2).

**Table 2:** Emergency ambulance service and hospital use by patients in the 6 months prior to eligibility for inclusion in strategies to manage emergency ambulance telephone callers with sustained high needs: an evaluation using linked data (STRETCHED) protocol.

Healthcare service	Intervention n = 550		Control n = 633	
Emergency ambulance service use (per patient): mean (±SD)				
Emergency calls	11.61	(17.13)	15.48	(88.93)
In-hours^a^ emergency calls	3.18	(5.77)	4.19	(17.76)
Out-of-hours^a^ emergency calls	8.42	(12.73)	11.22	(72.18)
Ambulance attendances	6.37	(7.06)	6.92	(14.52)
Ambulance conveyances	3.14	(4.39)	2.97	(3.67)
Hospital use				
Patients with at least 1 emergency^b^ admission: n (%)	351	(63.8%)	402	(63.5%)
Emergency^b^ admissions per patient: mean(±SD)	2.01	(2.80)	1.76	(2.27)
Patients with at least one ED attendance: n (%)	458	(83.3%)	505	(79.8%)
ED attendance per patient: mean (±SD)	5.10	(7.38)	4.40	(6.92)
Patients with at least 1 elective^c^ hospital admission: n (%)	90	(16.4%)	84	(13.3%)
Elective^c^ hospital admissions per patient: mean (±SD)	0.36	(2.71)	0.24	(1.18)

ED, emergency department.

a “In hours” calls include all 999 calls made between 800 and 1800 on weekdays, excluding UK bank holidays. Out of hours calls include all 999 calls outside these times (ie, calls made between 1800 and 0800, on weekends or bank holidays).

b Emergency admissions are unplanned admissions.

c Elective admissions are planned admissions.

### 3.3 Primary Outcomes

We did not detect a statistically significant difference between study arms for our composite primary outcome (OR, 1.159; 95% CI, 0.595, 2.255; *P*=.665), or its components: death, emergency admissions, ED attendances, and further emergency ambulance service calls (Table 3).

**Table 3:** The composite primary outcome and component indicators within the 6 months follow-up period.

Outcome	Intervention (n = 550)		Control (n = 633)		Adjusted comparison^a^	
					Difference (95% CI)	*P*-value
Composite indicator						
Mortality^b^: n (%)	58	(10.5%)	89	(14.1%)	OR, 0.713 (0.465-1.093)	.121
Mortality or emergency admission: n (%)	384	(69.8%)	446	(70.5%)	OR, 1.013 (0.748-1.372)	.933
Mortality or emergency admission or ED attendance: n (%)	464	(84.4%)	535	(84.5%)	OR, 1.005 (0.675-1.495)	.981
Mortality or emergency admission or ED attendance or emergency ambulance service call: n (%)	526	(95.6%)	601	(94.9%)	OR, 1.159 (0.595-2.255)	.665
Individual components						
At least 1 emergency admission recorded: n (%)	371	(67.5%)	422	(66.7%)	OR, 1.114 (0.831-1.492)	.470
At least 1 ED attendance: n (%)	423	(76.9%)	468	(73.9%)	OR, 1.088 (0.763-1.551)	.642
At least 1 emergency ambulance service call: n (%)	483	(87.8%)	545	(86.1%)	OR, 1.197 (0.794-1.805)	.391

a Models were adjusted for ambulance service, patient characteristics (age; gender; ethnicity, dichotomized to Caucasian/non-Caucasian because of low counts in some groups; Townsend Deprivation quintiles) and baseline service use (numbers of: emergency hospital admissions; emergency department presentations; and further emergency ambulance calls).

b All-cause mortality, within 6 months.

Most patients (95.3%) experienced at least 1 health care contact within the composite primary outcome during follow-up. While approximately 87% of patients made at least one further emergency ambulance service call, almost as many (84.4%) experienced at least one other (more serious) health care contact. Approximately 1 in 8 patients died within the 6-month follow-up (10.5% intervention; 14.1% control; and12.4% overall).

### 3.4 Secondary Outcomes

A higher proportion of intervention patients experienced an elective admission than control patients (OR, 1.802; 95% CI, 1.198, 2.711; *P*=.005), and they experienced more elective admissions per patient (incidence rate ratio, 1.720; 95% CI, 1.229, 2.407; *P*=.0.002) (Table 4). However, we found no statistically significant differences between arms for our other secondary outcomes.

**Table 4:** Further outcomes within the 6-month follow-up period.

Outcome	Intervention (n = 550)		Control (n = 633)		Adjusted comparison^a^	
					Difference (95% CI)	*P*-value
Recorded counts for primary outcome components						
Emergency admissions per patient: mean (±SD)	2.27	(3.31)	1.94	(2.85)	IRR, 1.152 (0.984-1.349)	.083
ED attendances per patient: mean (±SD)	5.96	(10.67)	5.13	(10.34)	IRR, 0.973 (0.848-1.115)	.702
Emergency ambulance service calls per patient: mean (±SD)	17.17	(28.65)	15.35	(26.43)	IRR, 1.062 (0.930-1.212)	.394
Further health care contacts						
Patients with at least one elective hospital admission: n (%)	81/550	(14.7%)	54/633	(8.5%)	OR, 1.802 (1.198-2.711)	.005
Number of elective hospital admissions: mean (±SD)	0.30	(1.65)	0.12	(0.48)	IRR, 1.720 (1.229-2.407)	.002
Patients with at least one outpatient appointment: n (%)	390/550	(70.9%)	428/633	(67.6%)	OR, 1.056 (0.767-1.455)	.737
Number of outpatient appointments: mean (±SD)	4.91	(9.28)	3.61	(5.43)	IRR, 1.046 (0.905-1.210)	.548
Ambulance attendances: mean (±SD)	9.24	(13.90)	7.75	(11.96)	IRR,1.050 (0.918-1.200)	.493
Ambulance conveyances: mean (±SD)	4.18	(7.49)	3.43	(5.39)	IRR, 1.055 (0.915-1.217)	.469
Further patient outcomes						
Patients declassified^b^: n (%)	538/550	(97.8%)	626/633	(98.9%)	OR, 0.619 (0.228-1.681)	.347
Declassifed patients subsequently meeting the frequent caller criteria: n (%)	179/538	(33.3%)	183/626	(29.2%)	OR, 1.075 (0.800-1.446)	0.631
Referred for case management^c^: n (%)	39/97	(40.2%)	n/a	n/a	n/a	n/a

IRR, incidence rate ratio; OR, odds ratio.

a Models were adjusted for ambulance service, patient characteristics (age; gender; ethnicity, dichotomized to Caucasian/non-Caucasian due to low counts in some groups; Townsend Deprivation quintiles) and baseline service use (numbers of: emergency hospital admissions; emergency department presentations; and further emergency ambulance calls).

b Patients no longer meeting the frequent caller criteria at some point during follow-up.

c Limited to intervention areas with data available on referral for case management.

Almost all patients (98.4%) no longer met the frequent caller threshold at some point during the follow-up period (ie, they had not made 5 or more calls in the previous month or ≥12 calls over the previous 3 months). Approximately one-third of these patients subsequently met the call threshold again, with no statistically significant difference observed between study arms.

We had only limited data on referrals to case management, and there was insufficient data to compare adverse events between study arms.

### 3.5 Goodness of Fit

Goodness of fit assessments were generally acceptable. The Box-Tidwell test showed a potential nonlinear relationship between the composite outcome and age. Sensitivity analyses considering age quintiles, age deciles, the natural logarithm of age, and age squared were all consistent with the primary analysis. Residual plots showed no sign of pronounced curvature but indicated 1 potential outlier. Removing the potential outlier substantially improved fit, as measured using the Hosmer-Lemeshow test and Cox and Snell pseudo-R^2^, but had little effect on reported results.

## 4 Limitations

STRETCHED protocol was a retrospective, observational study. All findings are associative in nature, not causal.

Participating ambulance services identified control sites which matched the corresponding intervention site as closely as possible. However, options were limited by the rapid and disparate spread of case management and other “high-intensity user” models of care.

We estimated sample sizes based on ambulance service data provided during study design. Although we almost reached the total estimated sample size, patient identification was dominated by 1 service, with more than half of intervention patients from AS4. STRETCHED protocol was not powered to detect effects at the service level, especially with such variation in patient identification.

We were not confident of achieving a reasonable questionnaire response rate, so STRETCHED protocol relied on EHR to obtain outcomes used to justify the provision of case management. This approach has efficiencies but excludes patient wellbeing or experience, so there may be benefits or harms not captured in our outcomes, or effects on patients that would need to be self-reported. Furthermore, EHR-based outcomes may be too blunt, and we could have missed a smaller impact on routine outcomes, while a longer follow-up period might have better captured effects of “behavioral change.”

Data availability issues affected some outcomes, notably those defined based on data provided directly by ambulance services. We only retrieved partial data on referral for and provision of case management and other non-NHS outcome data such as police arrests and convictions. Improved harmonization and completeness of data sets relevant to this patient group would facilitate further cross-agency evaluations and comparisons.

## 5 Discussion

We did not find any differences between arms for our primary outcome or its components. This matches existing evidence, which is mixed on whether case management for people who frequently use health care services reduces service use.^16^^,^^17^ Although there was a statistically significant difference in the number of elective admissions between arms (0.3 intervention vs 0.12 control), this difference may not be clinically meaningful, and most patients had no elective admission.

There is a common characterization – both within health care and in the media – that this population needs to increase their resilience or ability to self-manage their problems, which are often described as social, emotional or ‘not coping’. We found many patients calling with persistent clinical needs, sometimes compounded by challenging life circumstances and hardship or lack of family/community support.^16^ Although case management attempts to provide holistic care to people who make high use of emergency health services, in practice we have found, in common with other researchers in this area, the patient group, as defined in terms of ‘frequent caller’ categorization rather than operationally, to be somewhat transient, with declassification and subsequent reclassification common.^8^ Furthermore, this group includes a high proportion of patients who are very unwell, with high 6-month mortality and a high level of clinical needs which may not be well matched to the service provided. In other cases, problems may be intractable and not amenable to simple, short-term solutions.^10^ Given its heterogeneous nature, it is likely that improved strategies for the identification of individuals most likely to benefit are needed for case management to be successful.^18^

Supporting and treating people with complex needs involves building trust and working flexibly with individuals – as emergency ambulance service staff acknowledged – yet these service users felt they had no say in processes and decisions affecting them.^16^ There are intrinsic challenges when an emergency ambulance service – designed to provide an immediate and short-term curative response – becomes engaged with needs which are complex, long-term, and multifaceted. These often require care or basic human services such as housing. Responses are often on an ‘as needed’ basis and fail to address underlying issues. This tension is present in many aspects of emergency ambulance service work but is particularly apparent among the group of callers who meet the frequent caller threshold.

Services need to be provided in an accessible and relevant way to create genuine opportunities to reduce demand. For example, mental health issues can present as an inability to follow routine or attend structured support sessions yet be judged as failing lifestyle behaviors. Ambulance service staff have talked about challenges of engagement with service users; from the perspective of service users, the issue may not be so much about engagement as to do with access and availability.^5^^,^^16^

Our findings do not necessarily mean that case management never works better than usual care or is not worth providing, but we found expectations of its impact, based on advice and previous small-scale uncontrolled evaluations, to be unrealistic. The question of whether case management should be introduced or continue to be supported – in current or modified forms – for defined groups requires further study.

## Author Contributions

Conceptualization: AW, BE, PG, AJ, AK, AP, NR, HS. Investigation and Data Acquisition: RWA, TD, BE, BAE, TF, RF, IMG, AK, AP, NR, AR. Methodology & Data Analysis: AW, TD, JS, AT, HS. Writing - Original Draft: AW, TD, AK, AP, JS, BS, HS. Writing - Revision: All, AW takes responsibility for the final version. Funding Acquisition: AW, AE, BE, BAE, PG, AJ, AK, RP, AP, JS, AT, HS.

## Funding and Support

This research was funded by the UK’s National Institute for Health and Care Research (NIHR) through its Health and Social Care Delivery Research program. NIHR award reference: 18/03/02.

## Conflict of Interest

Alan Watkins reports financial support was provided by the National Institute for Health Research. All other co-authors report financial support was provided by the National Institute for Health Research. Alan Watkins reports a relationship with the National Institute for Health Research that includes: board membership. Helen Snooks reports a relationship with the National Institute for Health Research that includes: board membership. Nigel Rees reports a relationship with the National Institute for Health Research that includes: board membership. If there are other authors, they declare that they have no known competing financial interests or personal relationships that could have appeared to influence the work reported in this paper.
