# Supplementary material for: Effectiveness of Multidisciplinary “Case Management” Approaches to the Management of Patients Who Make High Use of the Emergency Ambulance Service: A Quasi-experimental Evaluation Using Linked Routine Records
Source: J Am Coll Emerg Physicians Open. 2026 Jul 13;7(5):100459. doi: 10.1016/j.acepjo.2026.100459 (PMC13382176; doi:10.1016/j.acepjo.2026.100459)
Supplement: Supplementary Material — 1 [file mmc1.docx]

Supplementary Appendix 1

Population demographics for STRETCHED study sites

| Ambulance Service^a^ | EEAS | | | | LAS | | | | WAST | | | | WMAS | | | |
| --- | --- | --- | --- | --- | --- | --- | --- | --- | --- | --- | --- | --- | --- | --- | --- | --- |
| Site status | Intervention | | Control | | Intervention | | Control | | Intervention | | Control | | Intervention | | Control | |
| Constituent units [CCG/s; LHB^b]^ | NE Essex; W Suffolk; Ipswich & E Suffolk | | West Essex; Hertfordshire Valleys; E & N Hertfordshire | | Lewisham | | Barnet | | Cardiff & Vale | | Aneurin Bevin | | Birmingham & Solihull | | Wolverhampton | |
| Main population centres | Ipswich; Bury St. Edmunds; Colchester | | Harlow; St. Albans; Stevenage | | Lewisham | | Barnet | | Cardiff; Barry; Penarth | | Newport; Caerphilly; Chepstow | | Birmingham; Solihull | | Wolverhampton | |
| Total population^c^ | 927,000 | | 1,414,000 | | 287,000 | | 365,000 | | 469,000 | | 557,000 | | 1,136,000 | | 254,000 | |
| Adults (18+) (%)^c^ | 748,000 | 80.7% | 1,098,000 | 77.7% | 222,000 | 78.4% | 281,000 | 77.0% | 373,000 | 79.5% | 443,000 | 79.5% | 861,000 | 75.8% | 195,000 | 76.8% |
| Number and proportion of adults:^c^ | | | | | | | | | | | | | | | | |
| Aged 65+ | 219,000 | 29.3% | 248,000 | 22.6% | 28,000 | 12.4% | 55,000 | 19.6% | 79,000 | 21.2% | 114,000 | 25.7% | 176,000 | 20.4% | 43,000 | 22.1% |
| Female | 385,000 | 51.5% | 57,000 | 51.9% | 120,000 | 53.3% | 148,000 | 52.7% | 194,000 | 52.0% | 229,000 | 51.7% | 448,000 | 52.0% | 101,000 | 51.8% |
| Non-white | 48,000 | 6.4% | 48,000 | 15.4% | 101,000 | 44.9% | 111,000 | 39.5% | 56,000 | 15.0% | 448,000 | 4.7% | 308,000 | 35.8% | 70,000 | 35.9% |
| Resident address in Townsend quintiles 4 & 5^d^ | 157,000 | 21.0% | 292,000 | 26.6% | 217,000 | 96.4% | 188,000 | 66.9% | 161,000 | 43.2% | 180,000 | 40.6% | 501,000 | 58.2% | 127,000 | 65.1% |

Notes
a: EEAS: East of England Ambulance Service; LAS: London Ambulance Service; WAST: Welsh Ambulance Services Trust; WMAS: West Midlands Ambulance Service
b: CCG: Clinical Commissioning Group, for EEAS, LAS & WMAS; LHB: Local Health Board, for WAST
c: Estimates from the 2021 UK Census, presented to the nearest thousand people
d: Townsend quintiles 4 & 5 are, respectively, the second most and most deprived quintiles.
