# Supplementary material for: Effectiveness of Multidisciplinary “Case Management” Approaches to the Management of Patients Who Make High Use of the Emergency Ambulance Service: A Quasi-experimental Evaluation Using Linked Routine Records
Source: J Am Coll Emerg Physicians Open. 2026 Jul 13;7(5):100459. doi: 10.1016/j.acepjo.2026.100459 (PMC13382176; doi:10.1016/j.acepjo.2026.100459)
Supplement: Supplementary Material 2 [file mmc2.docx]

Supplementary Appendix 2

Key features of UK usual care and case management within participating ambulance services

| **UK usual care** | **Case management** |
| --- | --- |
| **Aim** | |
| To reduce calls made by people classified as frequent callers, largely through redirecting the patient to other services and discouraging them from further calling. | To identify and manage triggers and causes that lead to the patient making frequent calls (e.g., adverse childhood events, mental health, drug and alcohol dependency, frailty) |
| **Delivery** | |
| Within-service management | Multi-disciplinary team (MDT); typically with representation from the police, ED, Out of Hours primary care, voluntary sector, social services, and other appropriate agencies. cross-sector case management.  MDTs provide a network of support for the patient and to address their needs through multi-agency working. Professionals see it as part of their role to support people who frequently access the ambulance service, police or ED. |
| **Care Model Elements** | |
| Clinical Support Officer (CSO, usually paramedic or nurse dedicated to working within the ambulance service Frequent Caller team) contacts patient by letter stating they have called the emergency ambulance service more than normal and should seek help from their GP; contact number provided within letter for patient to talk to ambulance service manager  CSO calls or sends letter to patient’s GP to make them aware patient is calling the emergency ambulance service frequently  If calls persist, CSO may contact other services to intervene and support patient  CSO writes individual care plan which is shared with the call centre clinical team. When the patient calls he/she is triaged to a clinician in the call centre rather than an ambulance being sent  If caller persists, he/she may be referred to the police and/or court | Clinical Support Officer (CSO, usually paramedic or nurse dedicated to working within the ambulance service Frequent Caller team) speaks to GP or practice manager to assess patient’s case and needs  Patient is discussed at monthly MDT meeting. Around 50 patients may be discussed, 10-15 of whom make high use of ambulance service.  Patient is interviewed to assess unmet needs  Patient is allocated to appropriate agency to lead on care planning and provision. A care plan is created and shared so that any agency contacted by the patient knows what has been agreed  If calls persist or patient has an antisocial behaviour order, the CSO visits the patient along with a police officer or representative from the ED. |
